# The combination of positive anti‑WDR1 antibodies with negative anti‑CFL1 antibodies in serum is a poor prognostic factor for patients with esophageal carcinoma

**DOI:** 10.3892/mi.2023.71

**Published:** 2023-01-31

**Authors:** Masaaki Ito, Satoshi Yajima, Takashi Suzuki, Yoko Oshima, Tatsuki Nanami, Makoto Sumazaki, Fumiaki Shiratori, Hao Wang, Liubing Hu, Hirotaka Takizawa, Shu-Yang Li, Yasuo Iwadate, Takaki Hiwasa, Hideaki Shimada

**Affiliations:** 1 Department of Clinical Oncology, Toho University Graduate School of Medicine, Tokyo 143-8541, Japan; 2 Department of Gastroenterological Surgery, Toho University School of Medicine, Tokyo 143-8541, Japan; 3 Stroke Center, The First Affiliated Hospital, Jinan University, Guangzhou, Guangdong 510630, P.R. China; 4 Department of Anesthesiology, Stroke Center, The First Affiliated Hospital and Health Science Center, Jinan University, Guangzhou, Guangdong 510630, P.R. China; 5 Port Square Kashiwado Clinic, Kashiwado Memorial Foundation, Chiba 260-0025, Japan; 6 Department of Neurological Surgery, Graduate School of Medicine, Chiba University, Chiba 260-8670, Japan

**Keywords:** WD repeat-containing protein 1, cofilin 1, esophageal carcinoma, antibody biomarker, overall survival

## Abstract

WD repeat-containing protein 1 (WDR1) regulates the cofilin 1 (CFL1) activity, promotes cytoskeleton remodeling, and thus, facilitates cell migration and invasion. A previous study reported that autoantibodies against CFL1 and β-actin were useful biomarkers for diagnosing and predicting the prognosis of patients with esophageal carcinoma. Therefore, the present study aimed to evaluate the serum levels of anti-WDR1 antibodies (s-WDR1-Abs) combined with serum levels of anti-CFL1 antibodies (s-CFL1-Abs) in patients with esophageal carcinoma. Serum samples obtained from 192 patients with esophageal carcinoma and other solid cancers. And s-WDR1-Ab and s-CFL1-Ab titers were analyzed using the amplified luminescent proximity homogeneous assay-linked immunosorbent assay. Compared with those of healthy donors, the s-WDR1-Ab levels were significantly higher in the 192 patients with esophageal, whereas these were not significantly higher in the samples from patients with gastric, colorectal, lung, or breast cancer. In 91 patients treated with surgery, sex, tumor depth, lymph node metastasis, stage and C-reactive protein levels were significantly associated with overall survival, as determined using the log-rank test, whereas the squamous cell carcinoma antigen, p53 antibody and s-WDR1-Ab levels tended to be associated with a worse prognosis. Although no significant difference was observed in the survival between the positive and negative groups of s-WDR1-Abs or s-CFL1-Abs alone in the Kaplan-Meier test, the patients in the s-WDR1-Ab-positive and s-CFL1-Ab-negative groups exhibited a significantly poorer prognosis in the overall survival analysis. On the whole, the present study demonstrates that the combination of positive anti-WDR1 antibodies with negative anti-CFL1 antibodies in serum may be a poor prognostic factor for patients with esophageal carcinoma.

## Introduction

Esophageal carcinoma is a rapidly progressive disease and is life-threatening even at the early stages. The therapeutic efficacy of esophageal carcinoma is less satisfactory than that of several other types of cancer; therefore, the detection and diagnosis at the early stage of carcinoma are indispensable for improving the therapeutic outcomes of patients (1).

The serum levels of several antigens and antibodies have long been measured using the serological analysis of recombinant tumor cDNA expression libraries (SEREX) method, a useful screening method for tumor markers (2). SEREX uses patient serum to immunoscreen cDNA libraries prepared from cancer specimens. By sequencing the isolated cDNA clones, the SEREX method is suitable as for the large-scale screening for tumor antigens. Large-scale SEREX screening has already identified numerous antibodies or antigens, such as trophoblast cell surface antigen 2(3), solute carrier family 2 member 1(4), striatin 4(5), LDL receptor related protein associated protein 1(6), proprotein convertase subtilisin/kexin type 9(7), cofilin 1 (CFL1) and β-actin (ACTB) (8). CFL1 can depolymerize F-actin in a pH-dependent manner and is also involved in lung, pancreatic, and gastric cancer invasion and metastasis (9-11).

WD repeat-containing protein 1 (WDR1; also known as actin-interacting protein 1) regulates cofilin activity, promotes cytoskeletal remodeling, and thus facilitates cell migration and invasion (12). The presence of autoantibodies against WDR1 has been found in the sera of patients who have suffered a transient ischemic attack using SEREX screening (13). The authors have previously reported that serum ACTB and CFL1 antibody titers were inversely associated with the overall survival of patients (8), suggesting the importance of actin polymerization and depolymerization in determining the prognosis. Thus, the present study aimed to evaluate the serum anti-WDR1 antibody (s-WDR1-Ab) titers in patients with various types of cancer compared with healthy donors. The overall survival was then compared among those with positive and negative s-WDR1-Ab and serum anti-CFL1 antibody (s-CFL1-Ab) titers.

## Patients and methods

### Collection of serum samples

The present study was approved by the Ethics Committee of Toho University Graduate School of Medicine (nos. A18103_A17052_A16035_A16001_26095_25024_24038_22047, M21038_20197_19213), Chiba University Graduate School of Medicine (no. 2018-320) (Japan) and Kashiwado Memorial Foundation (no. 2012-001). Serum samples were collected from patients who provided written formal informed consent. A total of 672 serum samples were obtained from cancer patients. These included 192 samples from patients with esophageal carcinoma, 96 samples from patients with gastric cancer, 192 samples from patients with colorectal cancer, 96 samples from patients with lung cancer and 96 samples from patients with breast cancer; all samples were obtained at the Toho University Omori Hospital (Tokyo, Japan) from June, 2010 to February, 2016. Serum samples from patients with esophageal carcinoma were collected prior to any treatments at Toho University, Omori Medical Center. Among these, 91 patients underwent radical surgery, comprising 70 males and 21 females. The median age of the operated esophageal carcinoma patients was 67 years. A total of 63 patients were candidates for neoadjuvant chemotherapy. Each patient was followed-up until June, 2018 or until death, whichever occurred first. All data regarding clinicopathological characteristics and prognoses were retrospectively obtained. Serum samples from 96 healthy donors (48 males, 48 females) were obtained by excluding residual samples at the Port Square Kashiwado Clinic, China, Japan.

### Preparation and purification of antigenic WDR1 and CFL1 proteins

cDNA clones for CFL1 and WDR1 were separated using SEREX screening from the λZAP II phage cDNA library for the human esophageal carcinoma cell line, T.Tn (3,4,8), and human aortic endothelial cells (14,15), respectively. Full-length cDNAs of WDR1 were recombined into pGEX-4T-1 (Cytiva). ECOS^TM^ competent *Escherichia coli* BL-21 cells (Nippon Gene, Co., Ltd.) were transformed with prokaryotic expression plasmids, pGEX-4T-1, pGEX-4T-1-WDR1 and pGEX-4T-1-CFL1 (Cytiva), and then cultured for 3 h in a 200 ml Luria broth containing 0.1 mM isopropyl β-D-thiogalactopyranoside (IPTG; FUJIFILM Wako Pure Chemical Corporation). The cells were lysed by sonication in BugBuster Protein Extraction Reagent (Merck), and GST, GST-WDR1 and GST-CFL1 proteins were purified by affinity chromatography using glutathione-Sepharose columns (Cytiva), as previously described (16-18).

### Measurement of s-WDR1-Ab and s-CFL1-Ab levels and conventional serum markers

Serum samples were obtained prior to any treatment, and centrifuged at 3,000 x g for 10 min at 25˚C, and stored at -80˚C. The s-WDR1-Ab and s-CFL1-Ab levels were measured using the amplified luminescence proximity homogeneous assay-linked immunosorbent assay (AlphaLISA) with WDR1 and CFL1. AlphaLISA was conducted using 384-well microtiter plates (white opaque OptiPlate™, PerkinElmer, Inc.) containing 2.5 µl 1/100-diluted sera and 2.5 µl GST, GST-WDR1, or GST-CFL1 (10 µg/ml) in AlphaLISA buffer [25 mM HEPES (pH 7.4), 0.1% casein, 0.5% Triton X-100, 1 mg/ml dextran-500 and 0.05% Proclin-300] following the manufacturer's instructions (PerkinElmer, Inc.) and as previously described (19). The reaction mixture was incubated at room temperature for 6-8 h. Subsequently, anti-human IgG-conjugated acceptor beads (2.5 µl of 40 µg/ml) and glutathione-conjugated donor beads (2.5 µl of 40 µg/ml) (PerkinElmer, Inc.) were added followed by incubation for a further 7-28 days at room temperature in the dark. Chemical emission was read on an EnSpire Alpha microplate reader (PerkinElmer, Inc.; http://www.perkinelmer.com/lab-solutions/resources/docs/GDE_ELISA-to-AlphaLISA.pdf) as previously described (5,15,20,21). By subtracting the alpha photon counts of the GST control from those of the GST fusion proteins, specific reactions were calculated.

The serum levels anti-p53 antibodies (s-p53-Abs) (22,23) and squamous cell carcinoma antigen (SCC-Ag) (24), which were often measured worldwide (22-24), were also measured using standard procedures. The cut-off values for s-p53-Abs was set at 1.3 IU/ml and those for SCC-Ag was set at and 1.5 ng/ml.

### Statistical analyses

Differences between the two variables were analyzed using Fisher's exact test. Corresponding differences between the three variables were determined using the Kruskal-Wallis test with Bonferroni's correction and the Mann-Whitney U-test. Receiver operating characteristic (ROC) curve analysis was used to determine the predictive qualities of putative disease markers, and cut-off values were determined to maximize the total sensitivity and specificity and the Youden index. Optimal cut-off values for serum antibody levels that affect overall survival were determined using X-tile 3.6.1 software (Yale University, New Haven, CT, USA), as previously described (25). The Kaplan-Meier method was used to analyze survival and survival curves were drawn. In addition, the survival distributions of two groups were compared using the log-rank test. Statistical analyses were performed using EZR software (https://www.jichi.ac.jp/saitama-sct/SaitamaHP.files/statmed.html) (version 1.55) (26). P<0.05 was considered to indicate a statistically significant difference.

## Results

### Identification of WDR1 and CFL1 using SEREX screening

WDR1 was identified using large-scale SEREX screening with the sera of patients with a transient ischemic attack as an antigen recognized by serum IgG antibodies (13). The 172-1233 region of WDR1 cDNA (accession no. NM_017491) was isolated along with the coding sequence from 235-735. The cDNA was then recombined into pGEX-4T-1 and the recombinant GST-fused WDR1 protein was purified. As WDR1 regulates actin polymerization, GST-CFL1 protein was also purified as previously described (8).

### Comparison of s-WDR1-Ab levels between patients with solid cancers and healthy donors

**Figure 1:**
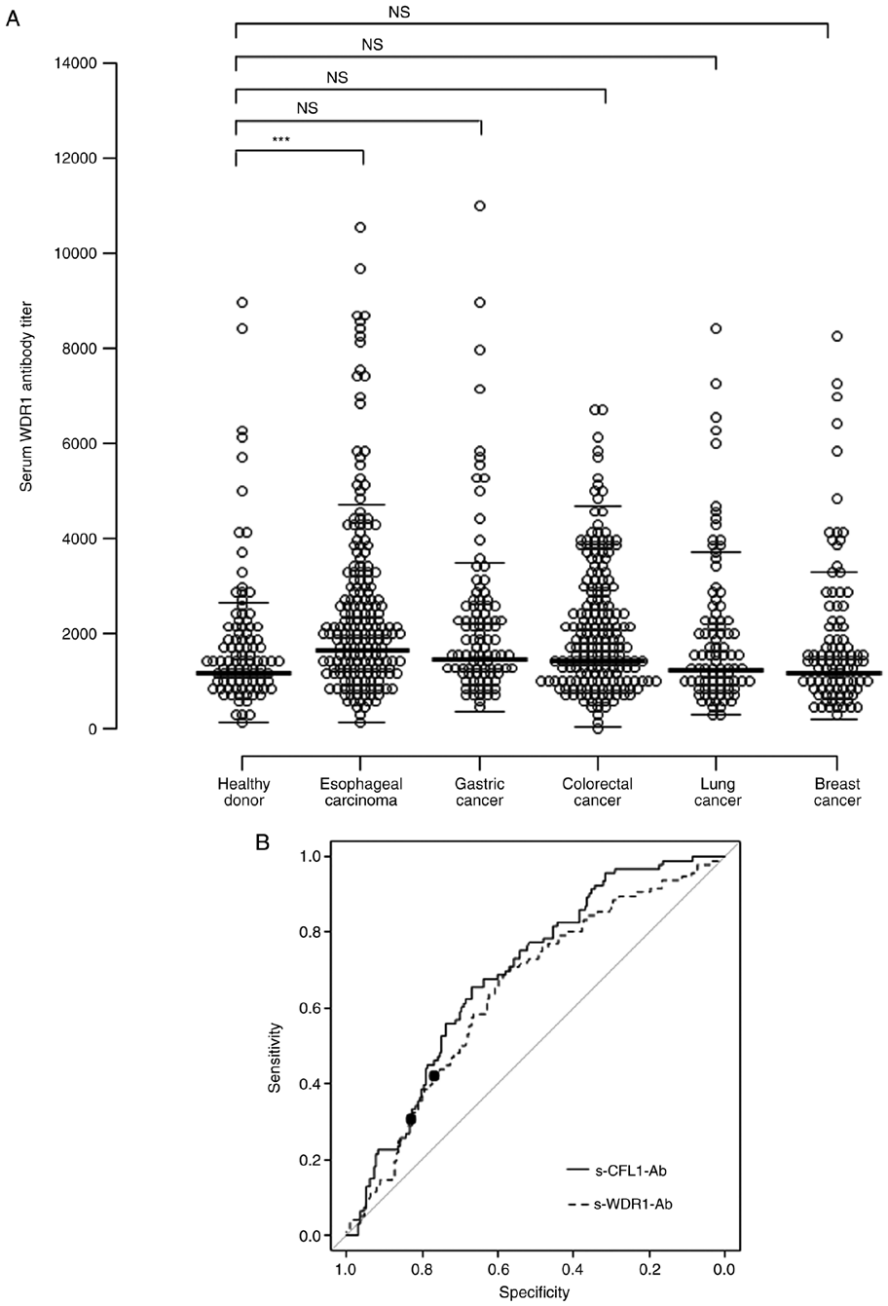
(A) Comparison of s-WDR1-Ab levels between healthy donors and patients with esophageal, gastric, colorectal, lung, and breast cancers examined using an amplified luminescence proximity homogeneous assay-linked immunosorbent assay (AlphaLISA). The s-WDR1-Ab levels are shown in a scatter dot plot. ^***^P<0.001; NS, not significant. (B) s-WDR1-Ab and s-CFL1-Ab levels using the ROC curve analysis between the alive and deceased cases in 192 patients with esophageal carcinoma. ROC curves for WDR1-Ab and CFL1-Ab are presented. The area under the ROC curve, 95% confidence interval, cut-off levels, specificity and sensitivity, and P-values are presented in Table SI. Closed circles indicate the positions with the highest sum of sensitivity plus specificity, i.e., the Youden index. The ROC curve analysis was used to calculate the P-values. WDR1, WD repeat-containing protein 1; CFL1, cofilin 1; s-WDR1-Ab, serum anti-WDR1 antibody; s-CFL1-Ab, serum anti-CFL1 antibody; ROC, receiver operating characteristic.

The s-WDR1-Ab level was measured with AlphaLISA using GST-WDR1 for antigens. The results revealed that the s-WDR1-Ab levels in patients with esophageal carcinoma were significantly higher than those from healthy donors (Fig. 1A; P<0.001). However, the s-WDR1-Ab levels in serum samples from patients gastric, colorectal, lung and breast cancers did not differ significantly from those in the serum of healthy donors, as determined using Bonferroni's correction.

ROC analysis resulted in an area under the ROC curve (AUC) for s-WDR1-Ab of 0.648 for esophageal carcinoma (Fig. 1B and Table SI). The sensitivity and specificity were 59.9 and 67.7%, respectively, with the determined cut-off value (1865) of the Youden index to maximize the sum of sensitivity and specificity. Setting the cut-off level of s-CFL1-Ab to 50718, the AUC value for s-CFL1-Ab was 0.694 with the sensitivity and specificity of 53.3 and 68.8%, respectively (Fig. 1B). Notably, the ROC curves of s-WDR-Ab and s-CFL-Ab were relatively similar.

### Association between the esophageal carcinoma patient clinicopathological parameters and overall survival

The associations between the overall survival and patient characteristics, including sex, age, tumor location, tumor depth, lymph node metastasis, stage, SCC-Ag, p53-Ab, s-WDR1-Ab, white blood cell count, as well as neutrophil, lymphocyte, hemoglobin, platelet, C-reactive protein (CRP) and albumin levels were evaluated using univariate analysis. The s-WDR1-Ab levels were divided into the high- and low-antibody groups (>1865/≤1865) based on the cut-off value calculated using X-tile analysis. Fisher's exact probability test revealed that the s-WDR1-Ab levels were significantly higher in male than those in female patients (Table I). However, the other parameters were not found to be significantly associated with the s-WDR1-Ab levels. As regards the stage, as the tumor stage progressed, the ratio of s-WDR1-Ab-positive cases vs. negative cases increased. The parameters of sex, tumor depth, lymph node metastasis and CRP levels were significantly associated with overall survival, as determined using the log-rank test (Table II), whereas the SCC-Ag, p53-Ab, and s-WDR1-Ab levels were only partly, but not significantly associated with the overall survival (P<0.066, 0.063 and 0.078, respectively). Since there was a significant difference in prognosis between males and females (Table II), the present study compared the prognosis of male and female patients in the high s-WDR1-Ab and low s-WDR1-Ab groups. The results did not reveal any significant difference, either in males or females (Fig. S1). Therefore, this result suggested that the difference in prognosis by sex appeared to have minimal relevance to the s-WDR1-Ab titer.

### Combined analysis of the s-WDR1-Ab and s-CFL1-Ab levels in association with patient survival

**Figure 2:**
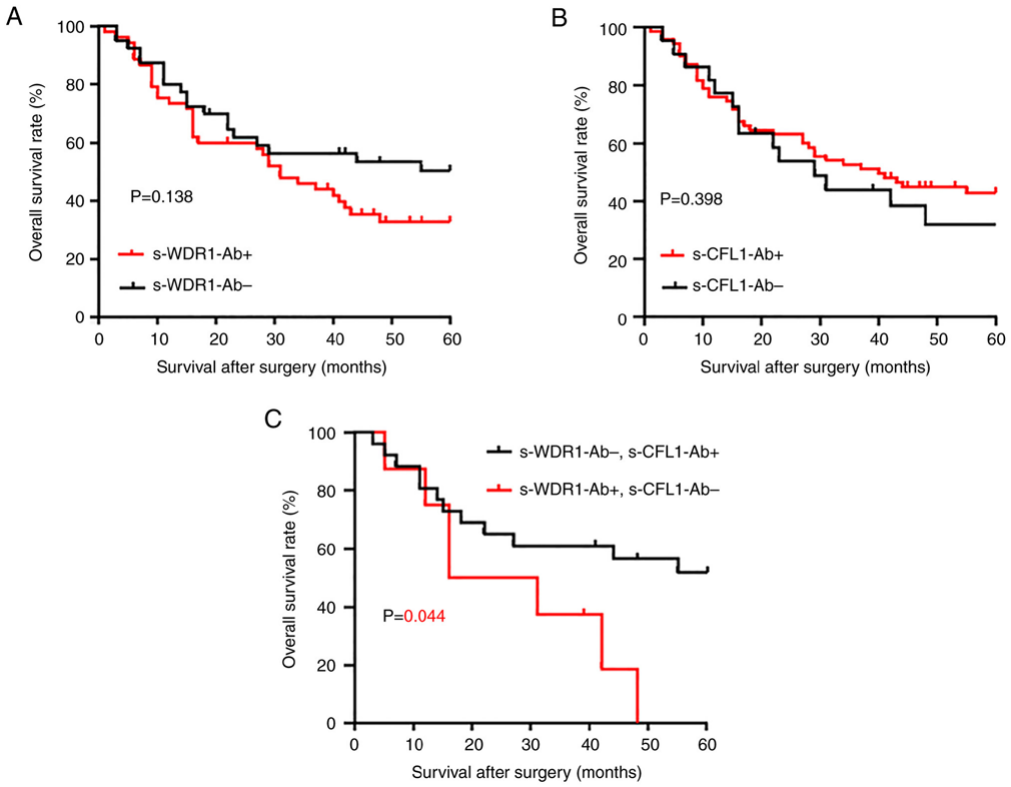
Comparison and combination of the overall survival of patients with esophageal carcinoma in the s-WDR1-Ab and s-CFL1-Ab groups. The levels of s-WDR1-Ab and s-CFL1-Ab were divided into two groups (positive, s-WDR1-Ab^+^ and s-XFL1-Ab^+^; negative, s-WDR1-Ab- and s-CFL1-Ab^-^) using the cut-off values calculated using the X-tile analysis. Overall survival was examined using the Kaplan-Meier method. (A) s-WDR1-Ab^+^ and s-WDR1-Ab^-^, (B) s-CFL1-Ab^+^ and s-CFL1-Ab^-^, and (C) s-WDR1-Ab^+^/s-CFL1-Ab^-^ and s-WDR1-Ab^-^/s-CFL1-Ab^+^. The log-rank test was used to evaluate any significant differences between each group. WDR1, WD repeat-containing protein 1; CFL1, cofilin 1; s-WDR1-Ab, serum anti-WDR1 antibody; s-CFL1-Ab, serum anti-CFL1 antibody.

To evaluate the prognostic characteristics of s-WDR1-Ab, survival curves were drawn using the Kaplan-Meier method. According to the cut-off level determined using the X-tile analysis, the s-WDR1-Ab and c-CFL1-Ab levels were divided into the positive and negative groups. Although no significant difference was observed in the survival of patients between the positive and negative groups of s-WDR1-Ab or s-CFL1-Ab levels alone (Fig. 2A and B), the combined group consisting of positive s-WDR1-Ab and negative s-CFL1-Ab exhibited a significantly poor prognosis in the overall survival analysis (Fig. 2C). Similarly, the group consisting of negative s-WDR1-Ab and positive s-CFL1-Ab exhibited a relatively favorable prognosis (Fig. 2C). After 50 months, approximately half of the patients in the latter group still survived, whereas those in the former group did not survive.

## Discussion

The present study found that the s-WDR1-Ab levels were significantly higher in patients with esophageal carcinoma than in healthy subjects (Fig. 1A). Moreover, the s-WDR1-Ab-positive group tended to have a poorer prognosis than the negative group (Fig. 2A). The combination of s-WDR1-Ab positivity and s-CFL1-Ab negativity revealed a significant difference vs. that of s-WDR1-Ab negativity and s-CFL1-Ab positivity (Fig. 2C). WDR1 expression has been shown to be upregulated in the highly metastatic gallbladder cancer cell line (GBC-SD18H) (27) and to be involved in the metastasis and poor prognosis of patients with hepatocellular carcinoma (28). Recently, the analysis of the role of WDR1 in tumors has focused on the aberrant expression of WDR1 in several tumors, including breast cancer, thyroid neoplasia, ovarian carcinoma and glioblastoma (29-32). In particular, Izawa *et al* (30) reported that anti-WDR1 antibody levels were significantly high in thyroid carcinoma and could be a novel serological biomarker for papillary thyroid carcinoma. The results of the present study also revealed significantly high levels of WDR1 antibody in esophageal carcinoma. The high expression levels of WDR1 protein may be one of the causes of the development of s-WDR1-Abs.

It is well known that the polymerization and depolymerization of actin microfilaments play indispensable roles in cell division, migration, invasion and metastasis. Thus, regulated polymerization and depolymerization may be required for cancer progression. As WDR1 enhances CFL1-mediated actin disassembly (33), the high expression of both WDR1 and CFL1 may synergistically disrupt cancer progression. Similar to the CFL1 activity, actin polymerization inhibitors, such as cytochalasin D and aplyronine A may be used as anticancer drugs (34,35). The low expression of CFL1, which cannot depolymerize an actin filament, may be compensated by the high expression of WDR1. This may account for the poor patient prognosis observed herein in the combined s-CFL1-Ab-negative and s-WDR1-Ab-positive groups (Fig. 2C).

Examining the expression levels of intracellular proteins in cancer tissues may be difficult. If the serum autoantibody levels reflect the intracellular antigenic protein expression levels, examining serum antibody levels is much easier and non-invasive. Some antigenic proteins can be leaked out from necrotic or apoptotic cancer cells but are hardly detectable because of their rapid degradation in the serum. Conversely, IgG antibodies are highly stable. Thus, antibody biomarkers are much more sensitive and stable to enable early detection and diagnosis. Although antibody biomarkers are not yet widely known, p53-Ab has been commonly used in clinical practice (22,23).

In conclusion, the present study demonstrated that serum anti-WDR1 antibody titers were significantly higher in patients with esophageal carcinoma than in healthy subjects. The combination of positive anti-WDR1 antibodies with negative anti-CFL1 antibodies may be a poor prognostic factor for patients with esophageal carcinoma.

## Supplementary Material

The overall survival of (A) male and (B) female patients as regards the levels of s-WDR1-Abs. Curves were drawn using Kaplan-Meier plotter. The log-rank test was used to determine significant differences between each group. WDR1, WD repeat-containing protein 1; s-WDR1-Ab, serum anti-WDR1 antibody.

Comparison of the s-WDR1-Ab and s-CFL1-Ab levels in healthy donors vs. those in 192 patients with esophageal carcinoma.

## Figures and Tables

**Table I:** Comparison of serum levels according to clinicopathological characters of the patients with esophageal carcinoma using univariate analysis.

Variable	WDR1-Ab >1865	WDR1-Ab ≤1865	P-value
Sex			
Male	24	46	0.012
Female	14	7	
Age, years			
>65	22	31	>0.999
≤65	16	22	
Tumor location			
Upper	5	9	0.771
Lower	33	44	
Tumor depth			
T1	15	14	0.254
T2-T4	23	39	
Lymph node metastasis			
N0	17	24	>0.999
N1	21	29	
Tumor stage			
0	6	3	
I	6	8	
II	8	17	
III	16	18	
IV	2	7	
SCC-Ag (ng/ml)			
>1.5	10	21	0.260
≤1.5	27	31	
p53-Ab (U/ml)			
>1.30	7	10	>0.999
≤1.30	29	43	
WBC (/µl)			
>8,000	4	7	0.757
≤8,000	34	46	
Neutrophils (%)			
>70	8	11	>0.999
≤70	30	42	
Lymphocytes (%)			
>35	6	11	0.597
≤35	32	42	
Hemoglobin (g/dl)			
>12	25	36	>0.999
≤12	13	17	
Platelets (/µl)			
>150,000	37	36	0.232
≤150,000	1	6	
CRP (mg/dl)			
>0.3	10	19	0.370
≤0.3	27	33	
Albumin (g/dl)			
>3.5	29	39	0.811
≤3.5	9	14	

Fisher's exact probability test was used for statistical analysis. Stage was determined using the Classification by the Japan Esophageal Society (36). As regards tumor location, the oral side from the tracheal bifurcation was defined as the ‘upper’ location and the anal side from the tracheal bifurcation was defined as the ‘lower’ location. WDR1, WD repeat-containing protein 1; WDR1-Ab, serum anti-WDR1 antibody; N0, no lymph node metastasis; N1, lymph node metastasis exists; SCC-Ag, squamous cell carcinoma antigen; WBC, white blood cell; CRP, C-reactive protein. There were some cases for which SCC-Ag, p53-Ab and CRP were not measured.

**Table II:** Univariate analysis of the risk factors associated with the overall survival of 91 patients with surgically treated for esophageal carcinoma.

	Univariate analysis P-value^a^
Sex	**0.046**
Male/Female	
Age, years	0.425
>65/≤65	
Tumor location	0.284
Upper/lower	
Tumor depth	**<0.001**
T1/T2-4	
Lymph node metastasis	**<0.001**
N^-^/N^+^	
Stage	**<0.001**
0, I, II/III, IV	
SCC-Ag (ng/ml)	0.066
>1.5/≤1.5	
p53-Ab (U/ml)	0.063
>1.30/≤1.30	
WDR1-Ab	0.078
>1865/≤1865	
WBC (/µl)	0.994
>8,000/≤8,000	
Neutrophils (%)	0.127
>70/≤70	
Lymphocytes (%)	0.498
>35/≤35	
Hemoglobin (g/dl)	0.095
≤12/>12	
Platelets (/µl)	0.194
≤150,000/>150,000	
CRP (mg/dl)	**<0.001**
>0.3/≤0.3	
Albumin (g/dl)	0.172
≤3.5/>3.5	

^a^P-values were obtained using the log-rank test. P-values in bold font indicate statistically significant differences (P<0.05). As regards tumor location, the oral side from the tracheal bifurcation was defined as the ‘upper’ location and the anal side from the tracheal bifurcation was defined as the ‘lower’ location. WDR1, WD repeat-containing protein 1; WDR1-Ab, serum anti-WDR1 antibody; N0, no lymph node metastasis; N1, lymph node metastasis exists; SCC-Ag, squamous cell carcinoma antigen; WBC, white blood cell; CRP, C-reactive protein.

## Data Availability

The datasets used and/or analyzed during the current study are available from the corresponding author on reasonable request.

## References

[1] Suzuki T, Yajima S, Okamura A, Yoshida N, Taniyama Y, Murakami K, Ohkura Y, Nakajima Y, Yagi K, Fukuda T (2022). Prognostic impact of carcinoembryonic antigen in 1822 surgically treated esophageal squamous cell carcinoma: Multi-institutional study of the Japan Esophageal Society. Dis Esophagus.

[2] Sahin U, Tureci O, Schmitt H, Cochlovius B, Johannes T, Schmits R, Stenner F, Luo G, Schobert I, Pfreundschuhet M (1995). Human neoplasms elicit multiple specific immune responses in the autologous host. Proc Natl Acad Sci USA.

[3] Nakashima K, Shimada H, Ochiai T, Kuboshima M, Kuroiwa N, Okazumi S, Matsubara H, Nomura F, Takiguchi M, Hiwasa T (2004). Serological identification of TROP2 by recombinant cDNA expression cloning using sera of patients with esophageal squamous cell carcinoma. Int J Cancer.

[4] Kuboshima M, Shimada H, Liu TL, Nakashima K, Nomura F, Takiguchi M, Hiwasa T, Ochiai T (2006). Identification of a novel SEREX antigen, SLC2A1/GLUT1, in esophageal squamous cell carcinoma. Int J Oncol.

[5] Ito M, Hiwasa T, Oshima Y, Yajima S, Suzuki T, Nanami T, Sumazaki M, Shiratori F, Funahashi K, Takizawa H (2021). Identification of serum anti-striatin 4 antibodies as a common marker for esophageal cancer and other solid cancers. Mol Clin Oncol.

[6] Sumazaki M, Shimada H, Ito M, Shiratori F, Kobayashi E, Yoshida Y, Adachi A, Matsutani T, Iwadate Y, Mine S (2020). Serum anti-LRPAP1 is a common biomarker for digestive organ cancers and atherosclerotic diseases. Cancer Sci.

[7] Ito M, Hiwasa T, Oshima Y, Yajima S, Suzuki T, Nanami T, Sumazaki M, Shiratori F, Funahashi K, Li SY (2021). Association of serum anti-PCSK9 antibody levels with favorable postoperative prognosis in esophageal cancer. Front Oncol.

[8] Ito M, Hiwasa T, Yajima S, Suzuki T, Oshima Y, Nanami T, Sumazaki M, Shiratori F, Li SY, Iwadate Y (2022). Low anti-CFL1 antibody with high anti-ACTB antibody is a poor prognostic factor in esophageal squamous cell carcinoma. Esophagus.

[9] Daryabari SS, Fathi M, Mahdavi M, Moaddab Y, Hosseinpour Feizi MA, Shokoohi B, Safaralizadeh R (2020). Overexpression of CFL1 in gastric cancer and the effects of its silencing by siRNA with a nanoparticle delivery system in the gastric cancer cell line. J Cell Physiol.

[10] Li X, Ma G, Guo W, Mu N, Wang Y, Liu X, Su L (2021). Hhex inhibits cell migration via regulating RHOA/CDC42-CFL1 axis in human lung cancer cells. Cell Commun Signal.

[11] Werle SD, Schwab JD, Tatura M, Kirchhoff S, Szekely R, Diels R, Ikonomi N, Sipos B, Sperveslage J, Gress TM (2021). Unraveling the molecular tumor-promoting regulation of cofilin-1 in pancreatic cancer. Cancers (Basel).

[12] Li J, Brieher WM, Scimone ML, Kang SJ, Zhu H, Yin H, von Andrian UH, Mitchison T, Yuan J (2007). Caspase-11 regulates cell migration by promoting Aip1-Cofilin-mediated actin depolymerization. Nat Cell Biol.

[13] Hu L, Liu J, Shimada H, Ito M, Sugimoto K, Hiwasa T, Zhou Q, Li J, Shen S, Wang H (2022). Serum anti-BRAT1 is a common molecular biomarker for gastrointestinal cancers and atherosclerosis. Front Oncol.

[14] Xu J, Wan P, Wang M, Zhang J, Gao X, Hu B, Han J, Chen L, Sun K, Wu J (2015). AIP1-mediated actin disassembly is required for postnatal germ cell migration and spermatogonial stem cell niche establishment. Cell Death Dis.

[15] Kagaya A, Shimada H, Shiratori T, Kuboshima M, Nakashima-Fujita K, Yasuraoka M, Nishimori T, Kurei S, Hachiya T, Murakami A (2011). Identification of a novel SEREX antigen family, ECSA, in esophageal squamous cell carcinoma. Proteome Sci.

[16] Konzok A, Weber I, Simmeth E, Hacker U, Maniak M, Muller-Taubenberger A (1999). DAip1, a dictyostelium homologue of the yeast actin-interacting protein 1, is involved in endocytosis, cytokinesis, and motility. J Cell Biol.

[17] Wang H, Zhang XM, Tomiyoshi G, Nakamura R, Shinmen N, Kuroda H, Kimura R, Mine S, Kamitsukasa I, Wada T (2018). Association of serum levels of antibodies against MMP1, CBX1, and CBX5 with transient ischemic attack and cerebral infarction. Oncotarget.

[18] Sugimoto K, Mori M, Liu J, Shibuya K, Isose S, Koide M, Hiwasa T, Kuwabara S (2021). Novel serum autoantibodies against β-actin (ACTB) in amyotrophic lateral sclerosis. Amyotroph Lateral Scler Frontotemporal Degener.

[19] Shimada H, Shiratori T, Yasuraoka M, Kagaya A, Kuboshima M, Nomura F, Takiguchi M, Ochiai T, Matsubara H, Hiwasa T (2009). Identification of makorin 1 as a novel SEREX antigen of esophageal squamous cell carcinoma. BMC Cancer.

[20] Machida T, Kubota M, Kobayashi E, Iwadate Y, Saeki N, Yamaura A, Nomura F, Takiguchi M, Hiwasa T (2015). Identification of stroke-associated-antigens via screening of recombinant proteins from the human expression cDNA library (SEREX). J Transl Med.

[21] Li SY, Yoshida Y, Kobayashi E, Adachi A, Hirono S, Matsutani T, Mine S, Machida T, Ohno M, Nishi E (2020). Association between serum anti-ASXL2 antibody levels and acute ischemic stroke, acute myocardial infarction, diabetes mellitus, chronic kidney disease and digestive organ cancer, and their possible association with atherosclerosis and hypertension. Int J Mol Med.

[22] Shimada H, Ochiai T, Nomura F (2003). Titration of serum p53 antibodies in 1,085 patients with various types of malignant tumors: A multiinstitutional analysis by the Japan p53 Antibody Research Group. Cancer.

[23] Shimada H, Takeda A, Arima M, Okazumi S, Matsubara H, Nabeya Y, Funami Y, Hayashi H, Gunji Y, Suzuki T (2000). Serum p53 antibody is a useful tumor marker in superficial esophageal squamous cell carcinoma. Cancer.

[24] Shimada H, Nabeya Y, Okazumi S, Matsubara H, Shiratori T, Gunji Y, Kobayashi S, Hayashi H, Ochiai T (2003). Prediction of survival with squamous cell carcinoma antigen in patients with resectable esophageal squamous cell carcinoma. Surgery.

[25] Camp RL, Dolled-Filhart M, Rimm DL (2004). X-tile: A new bio-informatics tool for biomarker assessment and outcome-based cut-point optimization. Clin Cancer Res.

[26] Kanda Y (2013). Investigation of the freely available easy-to-use software ‘EZR’ for medical statistics. Bone Marrow Transplant.

[27] Wang JW, Peng SY, Li JT, Wang Y, Zhang ZP, Cheng Y, Cheng DQ, Weng WH, Wu XS, Fei XZ (2009). Identification of metastasis-associated proteins involved in gallbladder carcinoma metastasis by proteomic analysis and functional exploration of chloride intracellular channel 1. Cancer Lett.

[28] Lambert AW, Pattabiraman DR, Weinberg RA (2017). Emerging biological principles of metastasis. Cell.

[29] Kim DH, Bae J, Lee JW, Kim SY, Kim YH, Bae JY, Yi JK, Yu MH, Noh DY, Lee C (2009). Proteomic analysis of breast cancer tissue reveals upregulation of actin-remodeling proteins and its relevance to cancer invasiveness. Proteomics Clin Appl.

[30] Izawa S, Okamura T, Matsuzawa K, Ohkura T, Ohkura H, Ishiguro K, Noh JY, Kamijo K, Yoshida A, Shigemasa C (2013). Autoantibody against WD repeat domain 1 is a novel serological biomarker for screening of thyroid neoplasia. Clin Endocrinol.

[31] Haslene-Hox H, Oveland E, Woie K, Salvesen HB, Wiig H, Tenstad O (2013). Increased WD-repeat containing protein 1 in interstitial fluid from ovarian carcinomas shown by comparative proteomic analysis of malignant and healthy gynecological tissue. Biochim Biophys Acta.

[32] Xu H, Chen Y, Tan C, Xu T, Yan Y, Qin R, Huang Q, Lu C, Liang C, Lu Y (2016). High expression of WDR1 in primary glioblastoma is associated with poor prognosis. Am J Transl Res.

[33] Talman AM, Chong R, Chia J, Svitkina T, Agaisse H (2014). Actin network disassembly powers dissemination of Listeria monocytogenes. J Cell Sci.

[34] Shoji K, Ohashi K, Sampei K, Oikawa M, Mizuno K (2012). Cytochalasin D acts as an inhibitor of the actin-cofilin interaction. Biochem Biophys Res Commun.

[35] Utomo DH, Fujieda A, Tanaka K, Takahashi M, Futaki K, Tanabe K, Kigoshi H, Kita M (2021). The C29-C34 parts of antitumor macrolide aplyronine A serve as versatile actin-affinity tags. Chem Commun (Camb).

[36] (2017). Japanese classification of esophageal cancer, 11th Edition: Part II and III. Esophagus.

