# Supplementary material for: The combination of positive anti‑WDR1 antibodies with negative anti‑CFL1 antibodies in serum is a poor prognostic factor for patients with esophageal carcinoma
Source: Med Int (Lond). 2023 Jan 31;3(2):11. doi: 10.3892/mi.2023.71 (PMC9983066; doi:10.3892/mi.2023.71)

Figure S1. The overall survival of (A) male and (B) female patients as regards the levels of s-WDR1-Abs. Curves were drawn using Kaplan-Meier plotter. The log-rank test was used to determine significant differences between each group. WDR1, WD repeat-containing protein 1; s-WDR1-Ab, serum anti-WDR1 antibody.

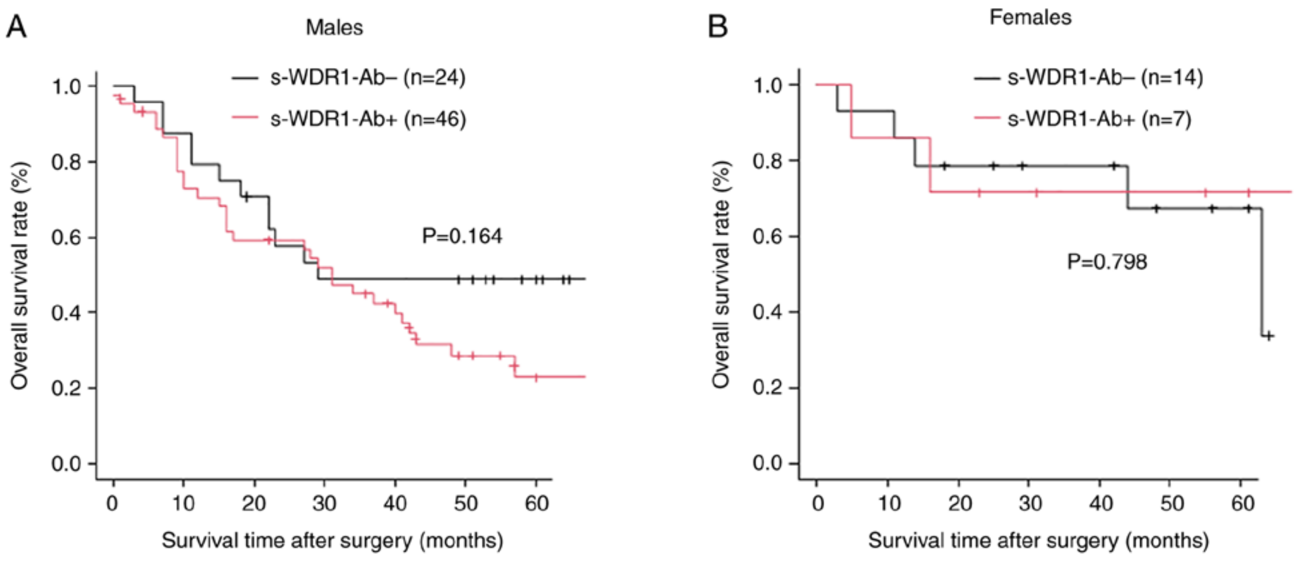

Supplement: The overall survival of (A) male and (B) female patients as regards the levels of s-WDR1-Abs. Curves were drawn using Kaplan-Meier plotter. The log-rank test was used to determine significant differences between each group. WDR1, WD repeat-containing protein 1; s-WDR1-Ab, serum anti-WDR1 antibody. [file Supplementary_Data1.pdf]
