# Supplementary material for: The combination of positive anti‑WDR1 antibodies with negative anti‑CFL1 antibodies in serum is a poor prognostic factor for patients with esophageal carcinoma
Source: Med Int (Lond). 2023 Jan 31;3(2):11. doi: 10.3892/mi.2023.71 (PMC9983066; doi:10.3892/mi.2023.71)
Supplement: Comparison of the s-WDR1-Ab and s-CFL1-Ab levels in healthy donors vs. those in 192 patients with esophageal carcinoma. [file Supplementary_Data2.pdf]

Table SI. Comparison of the s-WDR1-Ab and s-CFL1-Ab levels in healthy donors vs. those in 192 patients with esophageal carcinoma.

Results of statistical analysis	s-WDR1-Ab	s-CFL1-Ab
AUC	0.648	0.694
95% CI	0.590-0.703	0.636-0.747
P-value	<0.001	<0.001
Cut-off value (Youden index)	1865	50718
Sensitivity (%)	59.90	53.3
Specificity (%)	67.71	68.8

WDR1, WD repeat-containing protein 1; s-WDR1-Ab, serum anti-WDR1 antibody; CFL1, cofilin 1; s-CFL1-Ab, serum anti-CFL1 antibody; AUC, area under the curve; 95% CI, 95% confidence interval.
